# 100 Gy ^60^Co **γ**-Ray Induced Novel Mutations in Tetraploid Wheat

**DOI:** 10.1155/2014/725813

**Published:** 2014-05-20

**Authors:** Chuntao Yang, Jianshu Zhu, Yun Jiang, Xiaolu Wang, Mengxue Gu, Yi Wang, Houyang Kang, Xing Fan, Lina Sha, Haiqin Zhang, Pu Xuan, Yonghong Zhou

**Affiliations:** ^1^Triticeae Research Institute, Sichuan Agricultural University, Wenjiang, Chengdu, Sichuan 611130, China; ^2^Key Laboratory of Crop Genetic Resources and Improvement, Ministry of Education, Sichuan Agricultural University, Wenjiang, Chengdu, Sichuan 611130, China; ^3^Institute of Biological and Nuclear Technology, Sichuan Academy of Agricultural Sciences, Chengdu, Sichuan 610061, China

## Abstract

10 accessions of tetraploid wheat were radiated with 100 Gy ^60^Co **γ**-ray. The germination energy, germination rate, special characters (secondary tillering, stalk with wax powder, and dwarf), meiotic process, and high-molecular-weight glutenin subunits (HMW-GSs) were observed. Different species has different radiation sensibility. With 1 seed germinated (5%), *T. dicoccum* (PI434999) is the most sensitive to this dose of radiation. With a seed germination rate of 35% and 40%, this dose also affected *T. polonicum* (As304) and *T. carthlicum* (As293). Two mutant dwarf plants, *T. turgidum* (As2255) 253-10 and *T. polonicum* (As302) 224-14, were detected. Abnormal chromosome pairings were observed in pollen mother cells of both *T. dicoccoides* (As835) 237-9 and *T. dicoccoides* (As838) 239-8 with HMW-GS 1Ax silent in seeds from them. Compared with the unirradiated seed of *T. polonicum* (As304) CK, a novel HMW-GS was detected in seed of *T. polonicum* (As304) 230-7 and its electrophoretic mobility was between 1By8 and 1Dy12 which were the HMW-GSs of Chinese Spring. These mutant materials would be resources for wheat breeding.

## 1. Introduction

Human activities and natural calamities decreased the biological diversity and narrowed the genetic variability that limits crop breeding. Novel mutations in plants, which are crucial for improving resistance/tolerance to environmental stress, enhancing quality and yield traits, and facilitating the seed set of hybrid, have been created, such as in* Arabidopsis* [1], rice [2], maize [3], wheat [4], and some horticultural plants [5].

Since the 1970s, *γ*-rays, sodium azide, and ethyl methane sulfonate (EMS) have been used for wheat breeding [4]. Inducing mutation with ^60^Co *γ*-ray is an effective way and had bred some hexaploid wheat cultivars. Guinness/1322 (Bulgaria), for an example, was mutationally bred from Katya (a hexaploid wheat cultivar from Bulgaria) by 50 Gy ^60^Co *γ*-ray [6]. Compared with Katya, Guinness/1322 shows better lodging and shedding resistance, better ecological adaptability of drought tolerance, and higher productivity [7]. Inducing mutation with ^60^Co *γ*-ray was also used for tetraploid wheat breeding, but only two cultivars, Yavor (Bulgaria) and Implus (Turkey), were bred from durum wheat (AABB,  2*n* = 4*x* = 28) and different frequencies of induced mutations were observed under 100 Gy ^60^Co *γ*-ray [7, 8].

Tetraploid wheat (AABB, 2*n* = 4*x* = 28) distributes widely and adapts extensively to the environment and contains considerable wealth of genetic and morphological variation [9], such as high abilities of powdery mildew resistance in* Triticum dicoccoides *Körne [10], abundant genetic diversity of storage proteins in* T. dicoccoides *[10] and* Triticum turgidum* L. [11], valuable genes contributing to the grains per spike in* Triticum carthlicum *Nevski [12], dwarf genes in* Triticum polonicum* L. [13], and high content of gluten and tolerance to the saline in* Triticum durum* Desf. [14, 15]. Tetraploid wheat with AB genomes is important natural resources for breeding [16]. Therefore, creating novel mutation through radiation in tetraploid or hexaploid wheat may be an effective way for wheat breeding.

In the present study, 10 accessions of tetraploid wheat were radiated with 100 Gy ^60^Co *γ*-ray. Following the radiation, mutations of the agronomic traits, cytogenetics and high-molecular-weight glutenin subunits (HMW-GSs) were observed, which could be used for further selection and utilization of the radiated progenies.

## 2. Materials and Methods

### 2.1. Materials

All seeds of the accessions were deposited at Triticeae Research Institute, Sichuan Agricultural University, Sichuan, China. Information of the accessions was listed in Table 1.

### 2.2. Radiation

20 seeds of each accession were radiated with 100 Gy ^60^Co *γ*-ray at the Institute of Biological and Nuclear Technology, Sichuan Academy of Agricultural Sciences, China. Dose rate was 1.1 Gy/min, and unirradiated seeds were used as a control (CK).

### 2.3. Seed Germination

Respective 20 radiated and CK seeds of each accession were exposed with 4°C for 24 hours and germinated with 25°C. The germination energy (percentage of the seeds germinated in 10 days) and germination rate were calculated as follows:

(1) $$\begin{array}{c} \text{Germination}\;\text{rate}=\frac{\text{Number}\;\text{of}\;\text{germinated}\;\text{seeds}}{\text{Total}\;\text{number}\;\text{of}\;\text{seeds}\;\left(20\right)}\times 100\%. \end{array}$$

### 2.4. Agronomic Characters Identification

Radiated seedlings and CK ones were planted in the field. Agronomic characters including plant height, tiller number, seed set, and other special characters (secondary tillering, stalk with wax powder, and dwarf) were observed.

### 2.5. Meiotic Analysis

Young spikes were fixed in Carnoy's solution II (ethanol : chloroform : acetic acid = 6 : 3 : 1 V/V) and stored at 4°C. The pollen mother cells were stained with improved phenol fuchsin. Observations of the chromosome pairing of meiosis were made and documented with an Olympus BX-51 microscope coupled with a Photometric SenSys CCD camera. 60 cells of each accession were counted to confirm the pairing in the meiotic process.

### 2.6. High-Molecular-Weight Glutenin Subunits (HMW-GSs)

HMW-GSs of radiated and CK seeds were analyzed according to the method of Wan et al. [17]; eight seeds were tested in every single plant. The HMW-GSs of Chinese Spring (Null, 7 + 8, 2 + 12) were used as marker.

## 3. Results 

The results of seed germination were shown in Table 2. Among all the 10 accessions,* Triticum dicoccum *Schrank (PI434999) with only 1 seed germinated (5%) is the most sensitive to this dose of radiation.* T. polonicum* (As304) and* T. carthlicum *(As293) are 35% and 40% of germinations, respectively. The germinated ratio of the other accessions varied from 80% to 95%.

**Figure 1:**
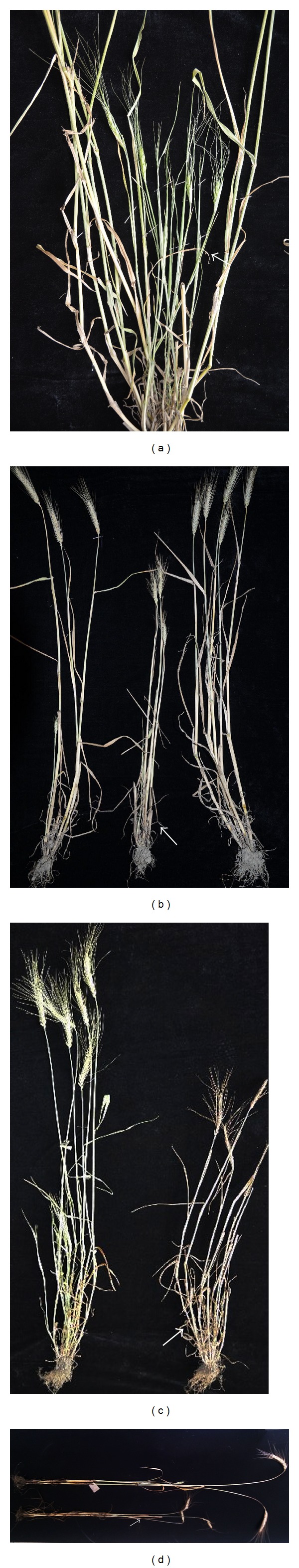
The special agronomic characters of the materials under radiation treatment. (a)* T. dicoccoides *(As838) 239-7, with 11 secondary tillerings and the after stalk with wax powder (arrowed), (b)* T. turgidum* (As2255) 253-10, dwarf (arrowed), (c)* T. carthlicum* (As293) 250-1, plant senescence (arrowed), and (d)* T. polonicum* (As302) 224-14, dwarf (arrowed).

The agronomic characters of all the radiated accessions were observed in the field. The results of varied agronomic characters of 4 radiated plants were shown in Table 3 and Figure 1. Compared with CK,* T. dicoccoides *(As838) 239-7 had 11 secondary tillerings and stalk with wax powder (Figure 1(a)). Dwarfs were observed in both* T. turgidum* (As2255) 253-10 (Figure 1(b)) and* T. polonicum *(As302) 224-14 (Figure 1(d)). Average height of* T. turgidum* (As2255) CK is 103.2 ± 2.5 cm, while that of* T. turgidum* (As2255) 253-10 is 68.5 cm. Average height of* T. polonicum *(As302) CK is 145.7 ± 5.9 cm, while that of* T. polonicum *(As302) 224-14 is 98.1 cm.* T. carthlicum *(As293) 250-1 was senescence, withered before harvest (Figure 1(c)). No other mutational agronomic characters were observed in other plants.

**Figure 2:**
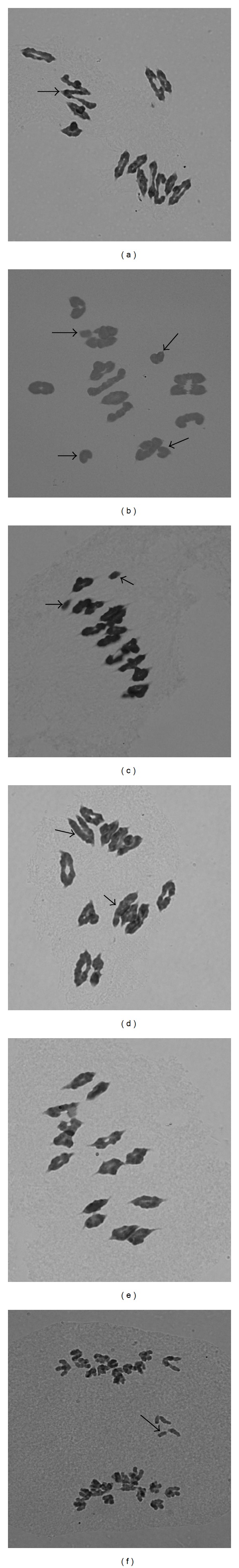
Chromosome pairing at metaphase I in the pollen mother cells. (a)* T. dicoccoides *(As835) 237-9, with a quadrivalent (arrowed), (b)* T. dicoccoides *(As835) 237-11, with 4 univalents (arrowed), (c)* T. dicoccoides *(As838) 239-7, with 2 univalents (arrowed), (d)* T. dicoccoides *(As838) 239-8, with 2 trivalents (arrowed), (e)* T. durum* (As781), with 14 rings of the chromosomes, and (f)* T. polonicum* (As304) 230-7, with lagging chromosomes (arrowed).

Chromosome pairing in meiosis of the plants with varied agronomic characters and some other radiated plants were also observed (Table 4). Univalents, trivalents, quadrivalents, and lagging chromosomes in meiosis were detected in few cells of the observed accessions. Quadrivalent was observed in* T. dicoccoides *(As835) 237-9 (Figure 2(a)). Univalents were observed in* T. dicoccoides *(As835) 237-11 (Figure 2(b)) and in* T. dicoccoides *(As838) 239-7 (Figure 2(c)). Trivalent in* T. dicoccoides* (As838) 239-8 was observed (Figure 2(d)). The normal chromosome pairing was shown in Figure 2(e). Lagging chromosomes were observed in* T. polonicum* (As304) (Figure 2(f)). Chromosome pairing results were as follows: radiation treatment had no effect on meiosis of 3 individuals with varied agronomic characters,* T. carthlicum *(As293) 250-1,* T. polonicum* (As302) 224-14 and* T. turgidum* (As2255) 253-10. Their chromosome pairing were 2*n* = 28 = 13.27II (ring) + 0.73II (rod), 2*n* = 28 = 10.56II (ring) + 3.43II (rod) and 2*n* = 28 = 13.05II (ring) + 0.95II (rod), respectively. Meanwhile, chromosome pairing of* T. dicoccoides *(As838)239-7 with 2*n* = 28 = 0.44I + 12.26II (ring) + 1.32II (rod) + 0.08III, exhibited a trait of 11 secondary tillerings and stalk with wax powder. The interference of chromosome pairing were also observed in radiated plants* T. dicoccoides *(As835) 237-11 (2*n* = 28 = 0.39I + 12.44II (ring) + 1.36II (rod)) and* T. dicoccoides *(As835) 237-9 (2*n* = 28 = 0.44I + 11.79II (ring) + 1.05II (rod) + 0.21III + 0.53IV) with 1Ax silence in seed numbered* T. dicoccoides *(As835) 237-9-5.* T. dicoccoides *(As838) 239-8 (2*n* = 28 = 1I + 12.32II (ring) + 0.64II (rod) + 0.36III) with 1Ax silence in seed numbered* T. dicoccoides *(As838) 239-8-2 and* T. polonicum* (As304) 230-7 2*n* = 28 = 0.10I + 12.11II (ring) + 1.41II (rod) + 0.10III + 0.18IV with a novel HMW-GS observed in seed numbered* T. polonicum* (As304) 230-7-1. Their chromosome pairings of meiotic process are abnormal, compared to the meiosis of the CK ones.

**Figure 3:**
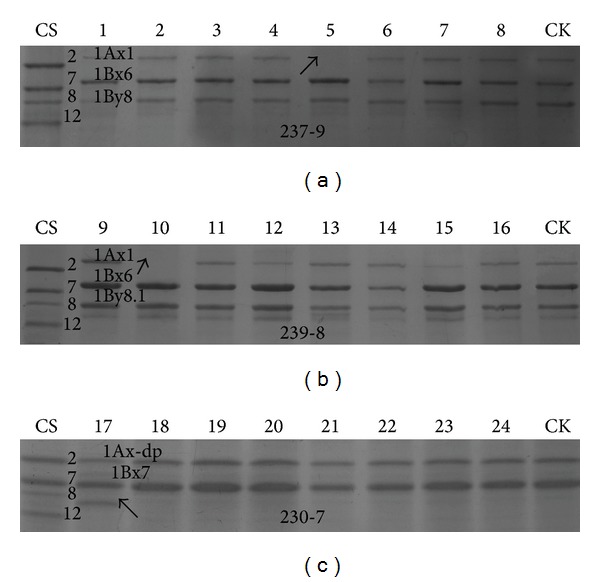
The SDS-PAGE of high-molecular-weight glutenins (HMW-GS). (a) CS (Chinese Spring), 1–8: seeds of plant* T. dicoccoides *(As835) 237-9; the absence of 1Ax1 was marked by an arrow; CK: no radiated* T. dicoccoides *(As835), (b) CS (Chinese Spring), 9–16: seeds of plant* T. dicoccoides *(As838) 239-8; the absence of 1Ax1 was marked by an arrow; CK: no radiated* T. dicoccoides *(As838), and (c) CS (Chinese Spring), 17–24: seeds of plant* T. polonicum* (As304) 230-7; the novel HMW-GS was marked by an arrow; CK: no radiated* T. polonicum* (As304).

HMW-GSs of eight randomly selected seeds of each single radiated plant of all the 10 tetraploid accessions were tested by SDS-PAGE. Three mutations were found. 1Ax was silent in* T. dicoccoides *(As835) 237-9-5 and* T. dicoccoides *(As838) 239-8-2 (Figures 3(a) and 3(b)). Compared with CK, a novel HMW-GS in* T. polonicum* (As304) 230-7-1 was detected whose electrophoretic mobility was between 1By8 and 1Dy12 which were the HMW-GSs of Chinese Spring (Figure 3(c)).

## 4. Discussion

Different species has different suitable dose of radiation intensity, such as 300 to 700 Gy ^60^Co *γ*-ray in* Sorghum bicolor *(L.) Moench [18], less than 200 Gy in* Roegneria *[19]. Suitable dose of radiation is various among different species in same genus [19]. In the present study, 100 Gy ^60^Co *γ*-ray differently induced mutations in the tetraploid wheat accessions. The results of germination energy and germination rate suggest that* T. dicoccum *(PI434999) is the most sensitive to the treatment and this dose of radiation is lethal dose to it. As to most tetraploid wheat, 100 Gy ^60^Co *γ*-ray radiation is an insufficient dose to induce mutation.

Chromosomal translocation, chromosome breakage, and deletions in chromosome which came from radiation mutation may lead to defects in chromosome pairing [20]. In this study, the abnormal chromosome pairings in meiotic process, such as univalents, trivalents, quadrivalents, and lagging chromosomes, were observed in pollen mother cells of some radiated plants, suggesting that 100 Gy ^60^Co *γ*-ray might create some mutations at chromosome level. Univalents were observed in* T. dicoccoides *(As838) 239-7 with 11 secondary tillerings and stalk with wax powder. Meanwhile, quadrivalent in* T. dicoccoides *(As835) 237-9, trivalents in* T. dicoccoides* (As838) 239-8, and lagging chromosomes in* T. polonicum *(As304) 230-7 were observed with HMW-GS mutations. Thus, the abnormal chromosome pairing of meiotic process reflected radiation mutations. Meiotic process observation could be used as a tool for mutation identification at wheat earing stage.

HMW-GSs are important storage proteins in wheat and its related species and 10% of endosperm proteins are HMW-GSs [21–23]. Theoretically, tetraploid wheat should contain 4 different HMW-GSs, 1Ax, 1Ay, 1Bx and 1By [23], but only one or two, no more than three subunits, were expressed due to gene silencing. Different HMW-GSs combinations have different effect on flour quality [23]. In the present study, compared with CK, 1Ax was silent in* T. dicoccoides *(As835) 237-9-5 and* T. dicoccoides *(As838) 239-8-2. HMW-GS gene silencing might be caused by specific nucleotide substitutions in the promoter region [21] and single repeat changes or repeat indels or large deletions in codon region [22, 23]. A novel HMW-GS was detected in* T. polonicum *(As304) 230-7-1 and its electrophoretic mobility was between 1By8 and 1Dy12 which were the HMW-GSs of Chinese Spring. Single nucleotide mutation or repeat deletions could restore the expression of genes; homoeologous recombination might be a novel pathway for allelic variation or molecular evolution of HMW-GSs [22, 24]. The mechanism of mutations in HMW-GS is under research.

Dwarf genes were found to be affecting architecture of rice plant [25]. GID1 gibberellin receptors affect the plant height of* Arabidopsis* [26]. 10 dwarfing genes/alleles have been discovered from tetraploid wheat [13, 27–29]. Associating with an extreme dwarf trait, only a few dwarfing genes have been used for wheat breeding worldwide [30]. Digging new plant height reducinggene is more and more important for wheat dwarf breeding. In the present study, significant plant dwarf was observed in both radiated plants* T. polonicum *(As302) 224-14 and* T. turgidum* (As2255) 253-10. The average height of* T. polonicum* (As302) is 145.7 ± 5.9 cm, while the radiation mutation of* T. polonicum* (As302) 224-14 is 98.1 cm in height. A nature mutant dwarf accession of* T. polonicum* (As304) is 68 cm in height. Radiation may cause different dwarf gene and the effect of dwarf accumulated* T. turgidum* 253-10 shows an extreme dwarf trait.

Inducing mutations for genetic improvement in breeding resources has been successfully and widely used for plant breeding. Sodium azide, EMS, and *γ*-rays are major tools for mutation. Sodium azide was widely used for mutation and breeding in rice [31], barley [32], tomato [33], and maize [3] but was not an effective mutagen in* Arabidopsis *[34]. EMS mainly induced single nucleotide mutations in* Arabidopsis thaliana *[1], hexaploid wheat, and* Triticale *[35]. Inducing mutations through chromosome aberration and single nucleotide mutant enriched the gene banks of the species [33]. During the past fifty years, about 130 wheat cultivars bred from mutation have been widely produced in China [36]. In the present study, some novel mutations in several tetraploid wheat cultivars were induced by 100 Gy ^60^Co *γ*-ray, such as HMW-GS and dwarf trait, which could be used as resources for theoretical study and future wheat breeding.

## 5. Conclusion 

In the present study, 100 Gy ^60^Co *γ*-ray differently induced mutations in accessions of tetraploid wheat. Following the radiation the germinated ratio of the materials varied from 5% to 95% and this dose of radiation is lethal dose to* T. dicoccum* (PI434999). The effects of radiation on the meiotic process of pollen mother cells and HMW-GSs were observed. Univalents, trivalents, quadrivalents, and lagging chromosomes in meiosis were detected in few cells of the observed accessions. As to HMW-GS, 1Ax was silent in* T. dicoccoides *(As835) 237-9-5 and* T. dicoccoides *(As838) 239-8-2 and a novel HMW-GS was detected in* T. polonicum *(As304) 230-7-1 whose electrophoretic mobility was between 1By8 and 1Dy12 which were the HMW-GSs of Chinese Spring. Compared to the CK,* T. dicoccoides *(As838) 239-7 had 11 secondary tillerings and stalk with wax powder. Plant dwarfs were also observed; the height of the radiated* T. turgidum* (As2255) 253-10 is 68.5 cm and* T. polonicum* (As302) 224-14 is 98.1 cm. These mutations would be resources for the future wheat breeding.

## Figures and Tables

**Table 1:** Materials used in this study.

Species	Accession number	Ploidy	Genome	Origin
*Triticum carthlicum *Nevski	As293	2*n* = 4*x* = 28	AABB	Japan
*Triticum dicoccoides *Körne	As835	2*n* = 4*x* = 28	AABB	Israel
*Triticum dicoccoides *Körne	As838	2*n* = 4*x* = 28	AABB	Israel
*Triticum dicoccum *Schrank	PI434999	2*n* = 4*x* = 28	AABB	Bosnia and Herzegovina
*Triticum durum *Desf.	As781	2*n* = 4*x* = 28	AABB	America
*Triticum polonicum *L.	As302	2*n* = 4*x* = 28	AABB	Xinjiang, China
*Triticum polonicum *L.	As304	2*n* = 4*x* = 28	AABB	Xinjiang, China
*Triticum turanicum *Jakubz.	As2279	2*n* = 4*x* = 28	AABB	Xinjiang, China
*Triticum turgidum *L.	As2255	2*n* = 4*x* = 28	AABB	Beijing, China
*Triticum turgidum *L.	As313	2*n* = 4*x* = 28	AABB	Jianyang, China

**Table 2:** The results of the germination.

Species	Total number of seeds	Germination number	Germination rate (%)	Germination energy (%)
*T. carthlicum* (As293)	20	8	40	35
CK	20	19	95	95
*T. dicoccoides* (As835)	20	18	90	85
CK	20	20	100	100
*T. dicoccoides* (As838)	20	18	90	90
CK	20	20	100	95
*T. dicoccum* (PI434999)	20	1	5	5
CK	20	19	95	95
*T. durum* (As781)	20	17	85	80
CK	20	20	100	100
*T. polonicum* (As302)	20	16	80	75
CK	20	20	100	100
*T. polonicum* (As304)	20	7	35	35
CK	20	20	100	100
*T. turanicum* (As2279)	20	16	80	80
CK	20	20	100	100
*T. turgidum* (As2255)	20	17	85	85
CK	20	20	100	100
*T. turgidum* (As313)	20	19	95	95
CK	20	20	100	100

**Table 3:** Special agronomic characters of the materials under radiation treatment.

Species	Individuals	Plant height (cm)	Tiller number	Seed set	Special characters
*T. carthlicum* (As293)	250-1	85.4	11	0.01	Plant senescence
	CK	105.4 ± 1.8	8 ± 2	0.85	
*T. dicoccoides* (As838)	239-7	124.7	16	0.79	11 secondary tillerings, stalk with wax powder
	CK	113.7 ± 3.7	17 ± 1	0.51	
*T. polonicum* (As302)	224-14	98.1	4	0.39	Dwarf
	CK	145.7 ± 5.9	3 ± 0	0.8	
*T. turgidum* (As2255)	253-10	68.5	7	0.53	Dwarf
	CK	103.2 ± 2.5	5 ± 2	1.67	

**Table 4:** Chromosome pairing at metaphase I in the pollen mother cells of the materials with special traits after radiation treatment.

Species	Individuals	Number of cells observed	Number of chromosomes	Chromosomes pairing					
				I	II			III	IV
					Total	Ring	Rod		
*T. carthlicum* (As293)	250-1	60	28		14.00	13.27	0.73		
						11–14	0–3		
	CK	60	28		14.00	13.55	0.45		
						11–14	0–3		
*T. polonicum* (As302)	224-14	60	28		13.99	10.56	3.43		
						10–13	1–4		
	CK	60	28		14.00	11.08	2.92		
						10–13	1–4		
*T. turgidum* (As2255)	253-10	60	28		14.00	13.05	0.95		
						10–14	0–4		
	CK	60	28		14.00	13.26	0.74		
						10–14	0–4		
*T. dicoccoides* (As835)	237-9	60	28	0.44	12.84	11.79	1.05	0.21	0.53
				0–2		9–13	0–3	0-1	0-1
	237-11	60	28	0.39	13.80	12.44	1.36		
				0–4		10–14	0–3		
	CK	60	28		14.00	13.15	0.85		
						11–14	0–3		
*T. dicoccoides* (As838)	239-7	60	28	0.44	13.58	12.26	1.32	0.08	
				0–2		10–13	0–4	0-1	
	239-8	60	28	1	12.96	12.32	0.64	0.36	
				0–4		10–14	0–2	0-1	
	CK	60	28		14.00	13.36	0.64		
						12–14	0–2		
*T. polonicum* (As304)	230-7	60	28	0.10	13.52	12.11	1.41	0.10	0.18
				0-1		10–14	0–4	0-1	0-1
	CK	60	28		14.00	13.05	0.95		
						10–14	0–4		
